# Minimal percentage of dose received by 90% of the urethra (%UD90) is the most significant predictor of PSA bounce in patients who underwent low-dose-rate brachytherapy (LDR-brachytherapy) for prostate cancer

**DOI:** 10.1186/1471-2490-12-28

**Published:** 2012-09-14

**Authors:** Nobumichi Tanaka, Isao Asakawa, Kiyohide Fujimoto, Satoshi Anai, Akihide Hirayama, Masatoshi Hasegawa, Noboru Konishi, Yoshihiko Hirao

**Affiliations:** 1 Department of Urology, Nara Medical University, 840 Shijo-cho, Kashihara, Nara, 634-8522, Japan; 2 Department of Radiation Oncology, Nara Medical University, Nara, Japan; 3 Department of Pathology, Nara Medical University, Nara, Japan

**Keywords:** Prostate cancer, Brachytherapy, PSA bounce, Post-dosimetry, UD90 (%)

## Abstract

**Background:**

To clarify the significant clinicopathological and postdosimetric parameters to predict PSA bounce in patients who underwent low-dose-rate brachytherapy (LDR-brachytherapy) for prostate cancer.

**Methods:**

We studied 200 consecutive patients who received LDR-brachytherapy between July 2004 and November 2008. Of them, 137 patients did not receive neoadjuvant or adjuvant androgen deprivation therapy. One hundred and forty-two patients were treated with LDR-brachytherapy alone, and 58 were treated with LDR-brachytherapy in combination with external beam radiation therapy. The cut-off value of PSA bounce was 0.1 ng/mL. The incidence, time, height, and duration of PSA bounce were investigated. Clinicopathological and postdosimetric parameters were evaluated to elucidate independent factors to predict PSA bounce in hormone-naïve patients who underwent LDR-brachytherapy alone.

**Results:**

Fifty patients (25%) showed PSA bounce and 10 patients (5%) showed PSA failure. The median time, height, and duration of PSA bounce were 17 months, 0.29 ng/mL, and 7.0 months, respectively. In 103 hormone-naïve patients treated with LDR-brachytherapy alone, and univariate Cox proportional regression hazard model indicated that age and minimal percentage of the dose received by 30% and 90% of the urethra were independent predictors of PSA bounce. With a multivariate Cox proportional regression hazard model, minimal percentage of the dose received by 90% of the urethra was the most significant parameter of PSA bounce.

**Conclusions:**

Minimal percentage of the dose received by 90% of the urethra was the most significant predictor of PSA bounce in hormone-naïve patients treated with LDR-brachytherapy alone.

## Background

Several investigators have reported PSA (prostate-specific antigen) bounce, a transient PSA elevation that is frequently observed after low-dose-rate brachytherapy (LDR-brachytherapy) [1-10]. Although the factors that affect PSA fluctuation after LDR-brachytherapy are unclear, multiple factors including age, prostatitis due to radiation or urinary tract infection, acute urinary retention, laboratory error, instrumentation, ejaculation, radiation proctitis, and testosterone recovery after androgen deprivation therapy (ADT) are currently considered as etiologies of PSA bounce. Most importantly, it is difficult to differentiate between PSA bounce and biochemical failure, and this situation produces a dilemma for patients and physicians to determine whether the treatment has failed or not. Several cut-off values of PSA, such as 0.1, 0.2, and 0.4 ng/mL, have been used to define PSA bounce. Seventeen to 62% of patients who underwent LDR-brachytherapy showed a PSA bounce during the first 3 years after LDR-brachytherapy [1-10]. The incidence of PSA bounce in Japanese patients is reportedly similar to that in American patients [8,9].

We investigated PSA bounce rates after LDR-brachytherapy to elucidate independent predictors of PSA bounce in our series of patients.

## Methods

Two hundred patients who were clinically diagnosed with localized prostate cancer (cT1c-2cN0M0) and underwent LDR-brachytherapy between July 2004 and November 2008 and a minimum follow-up of 18 months were enrolled in this prospective study. The patients’ characteristics are shown in Table 1. The median age, PSA value at diagnosis, and follow-up period were 70 years (range: 51–80), 7.6 ng/mL (range: 3.1-32.1), and 38 months (range: 18–65), respectively. One pathologist (NK) with expertise in prostate cancer diagnosis reviewed the Gleason score of all biopsy specimens centrally. The PSA values (PSA; ST AIA-PACK PSA II) were measured at 1, 3, and 6 months after LDR-brachytherapy, and then every 6 months.

**Table 1:** Patients’ characteristics

	**Bounce (-)**	**Bounce (+)**	***p*value**
	**(n = 150)**	**(n = 50)**	
Age (year)			
mean, median (range)	69.2, 70.0 (55-80)	67.4, 68.5 (51-79)	0.132^§^
PSA at diagnosis (ng/mL)			
mean, median (range)	9.1, 7.8 (3.1-32.1)	7.8, 6.7 (3.7-16.7)	0.029^§^
10 or less	106	41	
10-20	37	9	
greater than 20	7	0	0.156^*^
biopsy Gleason score			
6 or less	91	37	
7	53	11	
8-10	6	2	0.210^*^
clinical T stage			
T1c	88	32	
T2a	50	15	
T2b	7	2	
T2c	5	1	0.904^*^
neo-Adjuvant/ Adjuvant			
none	102	35	
neo-Ad (+)	40	15	
Ad (+)	4	0	
neo-Ad (+), Ad (+)	4	0	0.417^*^
Combined EBRT			
yes	49	9	
no	101	41	0.050^*^
biochemical recurrence			
yes	10	0	
no	140	50	0.069^*^
Number of PSA measurement			
mean, median (range)	8.5, 8.0 (5-15)	10.3, 10.5 (6-14)	< 0.001^§^
Follow-up period (month)			
mean, median (range)	36.7, 36.0 (18-64)	46.0, 47.0 (20-65)	< 0.001^§^

^*^Chi-square test and ^§^student t-test.

The institutional review board of Nara Medical University approved this study, and informed consent was obtained from all patients after explaining the aim and methods of this study.

### Treatment

Among the 200 patients, 137 did not receive neoadjuvant or adjuvant androgen deprivation therapy (ADT), and 4 received both neoadjuvant and adjuvant ADT. The median period of neoadjuvant ADT was 6.0 months (range: 1 to 54 months), and the scheduled period of adjuvant ADT was 2 years. Of the remaining patients, 55 received only neoadjuvant ADT and 4 received only adjuvant ADT. One hundred and forty-two patients were treated with LDR-brachytherapy alone and 58 patients were treated with LDR-brachytherapy in combination with external beam radiation therapy (EBRT) (Table 1).

From July 2004 to April 2007, LDR-brachytherapy alone was performed at the prescribed dose of 145 Gy in 93 patients, and after May 2007 it was performed at the prescribed dose of 160 Gy in 49 patients. The prescribed dose was 110 Gy for the patients who received LDR-brachytherapy in combination with EBRT. The target portion of EBRT was determined one month after LDR-brachytherapy, and the patients received 45 Gy (in 25 fractions of 1.8 Gy per fraction) using a four-field box technique via 6–10 MV photon energy. The clinical target volume included both the whole prostate and one third of the proximal seminal vesicle.

From July 2004 to April 2007, LDR-brachytherapy was performed after preplanning by modified peripheral loading techniques using Mick’s applicator [11]. From May 2007 to October 2008, we introduced an intraoperative planning method, and thereafter used real-time planning and peripheral loading.

### Postdosimetric evaluation

The therapeutic planning and post-implant dosimetric evaluation were performed using the planning system, Interplant Version 3.3 (CMS, Inc., St. Louis, USA) from July 2004 to October 2008, and thereafter Variseed 8.0 (Varian Medical Systems, Palo Alto, CA, USA).

Post-implant CT scanning and post-implant dosimetric study was performed by one radio-oncologist (AI) at 1 month after LDR-brachytherapy. The dosimetric parameters analyzed in this study were minimal percentage of the dose received by 90% of the prostate gland (%D90), minimal dose (Gy) received by 90% of the prostate gland (D90), percentage prostate volume receiving 100% and 150% of the prescribed minimal peripheral dose (V100/150), minimal percentage of the dose received by 30% and 90% of the urethra (%UD30 and%UD90), minimal dose (Gy) received by 30% and 90% of the urethra (UD30 and UD90), and rectal volume (mL) receiving 100% of the prescribed dose (R100).

### Statistic analysis

To elucidate independent factors to predict PSA bounce in hormone-naïve patients who underwent LDR-brachytherapy alone, prostate volume at implantation, prostate volume at postdosimetry,%D90, D90, V100, V150, R100,%UD90, UD90,%UD30, and biologically effective dose (BED) were evaluated. The BED was calculated to evaluate an independent factor to predict PSA bounce, and an α/β ratio of 2 was used [12].

In this study, PSA bounce was defined as an elevation in the PSA value of 0.1 ng/mL or more compared to the previous lowest value (excluding the 1 month PSA value), followed by a decline to a level at or below the pre-bounce value. We used Phoenix definition (nadir + 2 ng/mL) as the definition of PSA failure [13]. Estimated PSA bounce-free rate was calculated by the Kaplan-Meier method. Cox proportional hazards model was used to determine predictive parameters of PSA bounce both in univariate and multivariate analysis (backward stepwise selection method). To analyze the differences in categorical parameters, the chi-square test was employed. Student’s *t*-test was used to evaluate the differences in continuous variables. ANOVA by Bonferroni’s procedure and Dunnett’s procedure were applied to intergroup comparisons of the incidence of PSA elevation of ≥0.1 ng/mL, as well as the incidence, time, height, and duration of PSA bounce among the 4 groups treated with monotherapy with or without neoadjuvant ADT and combination therapy with or without neoadjuvant ADT. All statistical analyses were performed using PASW Statistics 17.0 (SPSS Inc., Chicago, IL, USA). All *p* values below 0.05 were considered statistically significant.

## Results

Of all patients, 92 (46%) showed PSA elevation of 0.1 ng/mL or greater from a PSA nadir during the follow-up period. The mean duration from LDR-brachytherapy to the PSA elevation was 17.4 months (median: 17 months). Of these 92 patients, 10 showed PSA failure and 50 showed PSA bounce (54%). The mean time to PSA bounce was 16.4 months (median: 17 months). The mean height and duration of PSA bounce were 0.49 ng/mL (median: 0.29 ng/mL) and 9.8 months (median: 7.0 months), respectively. The estimated 3-year PSA bounce-free rate was 72.4%. The mean number of PSA measurements in patients without PSA bounce was 8.5 (median: 8) while that in patients with PSA bounce was 10.3 (median: 10.5). PSA was more frequently measured in patients with PSA bounce than in those without it (*p* < 0.001) (Table 1). Of these 50 patients with PSA bounce, 8 (16%) showed a second PSA bounce. The mean duration from LDR-brachytherapy to the second PSA bounce was 29.3 months (median: 29.5 months). The mean height and duration of the second PSA bounce were 0.47 ng/mL (median: 0.18 ng/mL) and 6.8 months (median: 6.0 months), respectively.

The mean PSA value at diagnosis was significantly higher in patients without PSA bounce than in patients with PSA bounce. The mean follow-up period and the number of PSA measurements in patients without PSA bounce were significantly shorter and smaller than those in patients with PSA bounce. There was no significant difference in the prostate volume at postdosimetry between patients with and without PSA bounce (Table 1).

Regarding postdosimetric parameters,%D90, V100, V150, and R100 showed no significant differences between patients with and without PSA bounce, while UD90 (%) and UD90 (Gy), showed a significantly higher value in patients with PSA bounce. Patients without PSA bounce showed a significantly higher BED (Table 2).

**Table 2:** Postdosimetric parameters (all patients: n = 200)

	**Bounce (-) (n=150)**	**Bounce (+) (n=50)**	**P value**
	**(mean ± SD)**	**(mean ± SD)**	**(t-test)**
Prostate volume at postdosimetry (mL)	27.3 ± 8.7	29.0 ± 8.9	0.233
%D90 (%)	110.6 ± 9.9	109.7 ± 9.1	0.577
D90 (Gy)	152.3 ± 24.5	153.3 ± 19.4	0.772
V100 (%)	93.7 ± 3.6	93.5 ± 3.6	0.700
V150 (%)	62.3 ± 10.4	62.1 ± 10.3	0.881
R100 (mL)	0.10 ± 0.19	0.08 ± 0.10	0.668
Urethral volume (mL)	0.53 ± 0.28	0.41 ± 0.23	0.004
%UD90 (%)	97.6 ± 12.8	101.8 ± 12.7	0.049
UD90 (Gy)	134.1 ± 24.5	142.5 ± 23.6	0.035
%UD30 (%)	140.2 ± 18.0	143.8 ± 22.3	0.256
Minimal urethral dose (Gy)	100.9 ± 21.6	111.3 ± 22.2	0.004
BED (Gy2)	188.5 ± 25.2	177.0 ± 23.2	0.005

**Figure 1:**
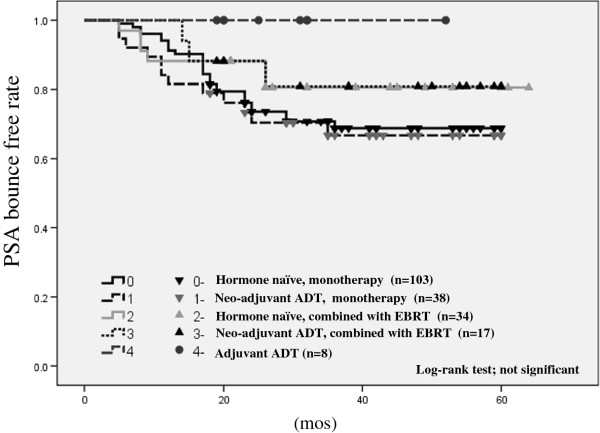
PSA bounce (0.1 ng/mL)-free rate in all patients stratified by treatment procedure (neoadjuvant/ adjuvant ADT and monotherapy/ combination with EBRT).

The incidence of PSA elevation of ≥0.1 ng/mL, the incidence of PSA bounce, the mean time to PSA bounce, the mean height of PSA bounce, and the estimated 3-year PSA bounce-free rate of each treatment group are summarized in Table 3. The incidence of PSA elevation of >0.1 ng/mL was significantly higher in the monotherapy with neoadjuvant ADT group than in the combination therapy without neoadjuvant ADT group (ANOVA; *p* = 0.006), whereas the incidence of PSA bounce showed no significant difference between the groups. The mean height of PSA bounce in the monotherapy without neoadjuvant ADT group was significantly higher than that in the monotherapy with neoadjuvant ADT group (ANOVA; *p* = 0.005), whereas the time to PSA bounce and PSA bounce duration showed no significant difference between the two groups. The estimated 3-year bounce-free rate of the monotherapy without neoadjuvant ADT, combination therapy without neoadjuvant ADT, monotherapy with neoadjuvant ADT, and combination therapy with neoadjuvant ADT groups were 68.8%, 80.6%, 66.7%, and 80.9%, respectively. Patients who underwent adjuvant ADT (n = 8) showed no PSA bounce during the follow-up periods (Figure 1). There were no significant differences in the estimated 3-year PSA bounce-free rates among the 4 groups (log-rank test).

**Table 3:** PSA bounce in each group

	**Hormone naive**		**Neo-adjuvant ADT**	
	**monotherapy**	**Combined EBRT**	**monotherapy**	**Combined EBRT**
	**(n=103)**	**(n=38)**	**(n=34)**	**(n=17)**
PSA elevation rate of 0.1 ng/mL or greater	51 (50%)	25 (66%)	9 (27%)	6 (35%)
Frequency of PSA bounce	29 (28%)	12 (32%)	3 (18%)	15 (18%)
Time to bounce (mos)	17.4, 17, (5-36)	14.8, 11.5 (5-35)	13.7, 8.5, (5-26)	18.3, 15, (14-26)
Mean, median, (range)				
Height (ng/mL)	0.51, 0.34, (0.13-1.74)	0.48, 0.26, (0.12-1.85)	0.19, 0.16, (0.10-0.40)	0.90, 0.68, (0.16-1.87)
Mean, median, (range)				
Duration (mos)	9.1, 6.0, (3-36)	11.8, 11.5, (2-31)	6.0, 6.0, (3-9)	15.3, 17.0, (7-22)
Mean, median, (range)				
3-yr bounce free rate (%)	68.8	80.6	66.7	80.9

### Subgroup analysis in hormone-naïve patients who underwent LDR-brachytherapy alone

The mean %UD90, UD90 and %D30% in patients who showed PSA bounce were significantly higher than those in patients without PSA bounce (Table 4). A univariate Cox proportional regression hazard model showed that age, %UD30, and %UD90 were independent predictors of PSA bounce after LDR-brachytherapy. Finally, %UD90 was the most significant parameter of PSA bounce in the multivariate Cox proportional regression hazard model (Table 5).

**Table 4:** Postdosimetric parameters (hormone-naïve, monotherapy: n = 103)

	**Bounce (-) (n=74)**	**Bounce (+) (n=29)**	**P value**
	**(mean ± SD)**	**(mean ± SD)**	**(t-test)**
Age (year)	68.8 ± 6.1	66.1 ± 7.7	0.060
PV at postdosimetry (mL)	27.9 ± 8.0	30.8 ± 8.2	0.109
%D90 (%)	110.1 ± 10.4	112.1 ± 8.0	0.338
D90 (Gy)	167.6 ± 17.6	164.3 ± 12.5	0.360
V100 (%)	93.6 ± 3.8	94.4 ± 2.7	0.272
V150 (%)	62.7 ± 8.9	65.5 ± 8.8	0.163
R100 (mL)	0.10 ± 0.20	0.10 ± 0.10	0.936
Urethral volume (mL)	0.55 ± 0.26	0.42 ± 0.23	0.019
%UD90 (%)	95.6 ± 12.1	104.7 ± 11.3	0.001
UD90 (Gy)	145.3 ± 18.2	153.3 ± 16.2	0.041
%UD30 (%)	140.2 ± 16.1	151.5 ± 21.8	0.005
Minimal urethral dose (Gy)	107.5 ± 19.3	119.1 ± 16.6	0.006
BED (Gy2)	177.5 ± 19.7	173.7 ± 13.9	0.354

**Table 5:** Univariate and multivariate analyses to predict PSA bounce (Cox proportional regression hazard model)

	**Univariate**	**Multivariate**	***p -*value**	**95% C.I.**
	***p -*value**	**Hazard ratio**		
Age	0.044			
%UD90(%)	0.004	1.037	0.007	1.010-1.064
%UD30 (%)	0.011			

*C.I* confidence interval.

## Discussion

PSA bounce, which is frequently observed after LDR-brachytherapy, is a curious phenomenon caused by an unknown mechanism. Reportedly, 17% to 62% of patients are diagnosed with PSA bounce using several definitions [1-10]. The median time to PSA bounce varied from 15 months to 26 months [1,2,4,6,7,9,10]. The median height of PSA bounce was 0.4 ng/mL to 0.8 ng/mL [1,2,4,6-9]. The median duration of PSA bounce was 6.8 months to 22.5 months [2,3,6,7]. In daily practice, doctors and patients are annoyed when PSA elevation shows biochemical recurrence or PSA bounce due to the comparatively high incidence and long duration of PSA bounce. In the present study, the incidence of PSA bounce, the median time to PSA bounce, the median height of PSA bounce, and the median duration of PSA bounce were 25%, 17 months, 0.29 ng/mL, and 7.0 months, respectively. The median height in our series was lower than that in previous studies. Presumably, it was caused by the definition of PSA bounce (cut-off value of 0.1 ng/mL).

In the subgroup analysis, the incidence of PSA elevation of ≥0.1 ng/mL was significantly higher in the monotherapy with neoadjuvant ADT group than the combination therapy without neoadjuvant ADT (*p* = 0.006) group. However, the incidence of PSA bounce was not significantly different in these four groups. This result was comparable to that of previous reports [5,6,10]. Moreover, the estimated 3-year bounce-free rates in these four groups showed no significant differences in this study.

The present study showed that %UD90, UD90, and BED were significantly different between patients with PSA bounce and those without PSA bounce, whereas age and prostate volume showed no significant difference among all patients. To avoid the influence of neoadjuvat/adjuvant ADT and EBRT, we evaluated the predictive parameters of PSA bounce in patients treated with LDR-brachytherapy alone without neoadjuvant/adjuvant ADT. In this group, %UD90, UD90 and %UD30 showed a significant difference between patients with PSA bounce and those without PSA bounce, while age showed a marginal difference (*p* = 0.06). Previous studies have reported that age, D90, isotope, and prostate volume were the significant predictive factors of PSA bounce [3-9]. On the other hand, age, %UD90 and %UD30 were significant predictive parameters of PSA bounce by univariate analysis using a Cox proportional regression hazard model in this study. Multivariate analysis indicated %UD90 as predictive parameters. The pathological reason why %UD90 affects the incidence of PSA bounce is unknown. It is most probable that a higher dose to the urethra causes a higher incidence of radiation-induced urethritis and prostatitis.

Various factors including age, radiation-induced proctitis, prostatitis (radiation-induced or infection), ejaculation, laboratory error, urinary retention, testosterone recovery after ADT, and instrumentation, are considered as etiologies of PSA bounce. Assessment strongly suggests that sexual activity, inflammatory stimulation and androgen manipulation are associated with PSA bounce. It is necessary to investigate the correlation between PSA bounce and the data of urine analysis, urodynamics study, and serum androgen level in the future.

Our present study has some limitations such as a small number of patients and a short follow-up period. Indeed, there were only 50 patients who showed PSA bounce out of the 82 patients with PSA elevation of ≥ 0.1 ng/mL after LDR-brachytherapy without PSA failure. The other 32 patients with PSA elevation did not reach the PSA nadir level during the follow-up period. Longer follow-up is necessary to confirm PSA bounce in this patient group. The difference in the prescribed monotherapy dose (145 Gy vs. 160 Gy) may also influence the incidence of PSA bounce. In this study, we did not evaluate this matter because the follow-up period of the patients who received a prescribed dose of 160 Gy was significantly shorter than that of the patients who received 145 Gy (data not shown). The definition of PSA bounce is also controversial. In this study, we adopted a cut-off value of 0.1 ng/mL. The results of studies with a longer follow-up period and a different cut-off value of PSA bounce are expected in the near future.

## Conclusions

There is no significant difference in the prevalence of PSA bounce between groups treated with LDR-brachytherapy alone and those treated with LDR-brachytherapy in combination with EBRT, regardless of neoadjuvant ADT. In hormone-naïve patients treated with LDR-brachytherapy alone, %UD90 was the most significant predictor of PSA bounce.

## Competing interests

The authors declare that they have no conflict of interest.

## Authors' contributions

All authors made substantial contributions to the acquisition and interpretation of data, critical revision of the manuscript for important intellectual content, and approved the final version for publication. YH, NK and MH made substantial contributions to the conception and design of the study. NT performed the statistical analysis. All authors read and approved the final manuscript.

## Pre-publication history

The pre-publication history for this paper can be accessed here:

http://www.biomedcentral.com/1471-2490/12/28/prepub
